# Responses of two dominant desert plant species to the changes in groundwater depth in hinterland natural oasis, Tarim Basin

**DOI:** 10.1002/ece3.7766

**Published:** 2021-06-16

**Authors:** Bilal Imin, Yue Dai, Qingdong Shi, Yuchuan Guo, Hao Li, Marhaba Nijat

**Affiliations:** ^1^ Key Laboratory of Oasis Ecology College of Resources and Environmental Science Xinjiang University Urumqi China; ^2^ Institute of Arid Ecology and Environment Xinjiang University Urumqi China

**Keywords:** Daryaboyi oasis, health status, Keriya River Basin, leaf carbon isotope, vegetation cover index, water use strategies

## Abstract

Groundwater is increasingly becoming a permanent and steady water source for the growth and reproduction of desert plant species due to the frequent channel cutoff events in arid inland river basins. Although it is widely acknowledged that the accessibility of groundwater has a significant impact on plant species maintaining their ecological function, little is known about the water use strategies of desert plant species to the groundwater availability in Daryaboyi Oasis, Central Tarim Basin. This study initially determined the desirable and stressing groundwater depths based on ecological and morphological parameters including UAV‐based fractional vegetation cover (FVC) images and plant growth status. Then, leaf δ^13^C values of small‐ and big‐sized plants were analyzed to reveal the water use strategies of two dominant woody species (*Populus euphratica* and *Tamarix ramosissima*) in response to the groundwater depth gradient. The changes in FVC and growth status of plants suggested that the actual groundwater depth should be kept at an appropriate range of about 2.1–4.3 m, and the minimum groundwater depth should be less than 7 m. This will ensure the protection of riparian woody plants at a normal growth state and guarantee the coexistence of both plant types. Under a desirable groundwater condition, water alternation (i.e., flooding and rising groundwater depth) was the main factor influencing the variation of plant water use efficiency. The obtained results indicated that big‐sized plants are more salt‐tolerant than small ones, and *T. ramosissima* has strong salt palatability than *P*. *euphratica*. With increasing groundwater depth, *P. euphratica* continuously decreases its growth status to maintain hydraulic efficiency in drought condition, while *T. ramosissima* mainly increases its water use efficiency first and decreases its growth status after then. Besides, in a drought condition, *T. ramosissima* has strong adaptability than *P*. *euphratica*. This study will be informative for ecological restoration and sustainable management of Daryaboyi Oasis and provides reference materials for future research programs.

## Graphical abstract

To ensure the protection of riparian woody plants at normal growth state and guarantee the coexistence of both plant types, the actual groundwater depth should be kept at an appropriate range of 2.1–4.3 m, and the minimum groundwater depth should be less than 7 m. *Populus* *euphratica* generally utilizes a conservative strategy with the reduction in water availability to withstand drought stress, while *Tamarix* spp. adopts a more optimized solution. The study also found that *Tamarix* spp. has a strong drought resistibility than *P. euphratica*.

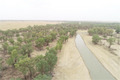

## 1 INTRODUCTION

Water resources are scarce but essential for fragile endorheic river–floodplain ecosystems in the middle Asian drylands (Karthe & Daniel, 2017; Schäfer et al., 2017). In these areas, desert riparian forests mainly consist of phreatophytes, *Populus euphratica* Oliv., and *Tamarix ramosissima,* which are primarily found in the floodplains of the catchments and are viewed as the lifeline of riparian zones (Han, Zhao, Feng, & Shi, 2015; Yu et al., 2021; Zhang, Zhou, Guan, et al., 2019) by providing critical habitats for various species and functioning as an "ecological shelter" against desertification (Ding et al., 2017). In desert riparian zones, groundwater is considered as a permanent and steady water source for native plant growth and reproduction due to the limited surface water flows and hyperarid climate conditions induced by severe drought events (Eamus et al., 2006; Evaristo & Mcdonnell, 2017; Li et al., 2020). Besides, in groundwater‐dependent arid ecosystems, plants could suffer from water stress when the groundwater depth is too deep, whereby soil water salinity could restrain plant growth when the water depth is too shallow (Maihemuti et al., 2021). Abiotic stress, such as water stress and salt stress, is one of the most deleterious environmental factors affecting plant growth, reproduction, and survivability during whole developmental stages (Galicia et al., 2020; Ma et al., 2020; Sun et al., 2010). Moreover, severe stress, including salt stress, may cease seed germination, decrease plant productivity (biomass) and growth status (vitality), and even lead to the death of the plants (Halik et al., 2019; Li, Si, Zhang, Gao, Luo, et al., 2019; Yu et al., 2018; Wang et al., 2013). Therefore, desert riparian plants are always sensitive to the fluctuations in the groundwater table associated with the variations in flood frequency, magnitude, duration, and seasonal discharge (Halik et al., 2019; Keram et al., 2019). These variations in the groundwater table may cause crucial constraints on the structure, distribution, and dynamics of riparian forests if they do not acclimate to this variability (Han, Zhao, Feng, Disse, et al., 2015; Li, Tong, et al., 2019; Schäfer et al., 2017; Ye et al., 2009). Several studies have reported that the contradiction between enlarged irrigated agriculture in the midstream area and degraded groundwater‐dependent natural ecosystem in the downstream sector has become increasingly prominent in the arid and semiarid endorheic river basins in NW China (Ding et al., 2017; Guo et al., 2017; Halik et al., 2019; Wang et al., 2014). As a result, ecosystem functions provided by the floodplain areas have been severely damaged and desertification has been extended because of these deteriorations (Mamat et al., 2018). Therefore, to meet this challenge, more urgent studies are required to investigate the desert riparian forests' response to the different groundwater status, which will provide informative references for ecological conservation and restoration in the dryland downstream oases.

The isotopic ratio of ^13^C/^12^C in plant tissue relative to a geologic standard is defined as foliar carbon isotopic composition (δ^l3^C) (Samuelson et al., 2018). Currently, δ^l3^C has been used to assess plant intrinsic water use efficiency (WUE) because there is a significant positive correlation between the two (Liu et al., 2020; Ma et al., 2018). WUE is also a critical indicator that indicates the ability of plants to adjust to water deficits, and it reflects the coupling relationship or trade‐offs between water and carbon cycles in the ecosystem (Bai et al., 2020; Verlinden et al., 2015). Foliar δ^l3^C not only offers a comprehensive insight into how plant species interplay with and respond to their biotic and abiotic environments but also reveals the resource acquisition strategies of plant species throughout the growth period (Cao et al., 2020; Fu et al., 2020; Ma et al., 2018). In addition, previous studies on the water use efficiency (WUE) of plant species have reported that foliar δ^l3^C is the best option for quantifying the WUE of plant species due to its advantages of high precision and low destructiveness (Bush et al., 2017; Marhaba et al., 2020; Zou et al., 2019). In recent decades, several studies have been conducted on the response of plant δ^l3^C to climate variables (Gatica et al., 2017; Liu et al., 2015; Sun et al., 2018), soil variables (Liu et al., 2020; Ogaya & Peñuelas, 2008; Verlinden et al., 2015), and habitat conditions (Song et al., 2019; Zou et al., 2019). Although few attempts have taken an interest in WUE of desert riparian forests in Tarim and Heihe River basins in arid regions of northwestern China, the studies have only taken into account single plant types or unique age structure (Ren et al., 2014; Si et al., 2015). Furthermore, the water use strategies of desert plants to the decreasing water availability in endangered terminal oases of arid inland river basins are still unknown.

**FIGURE 1:**
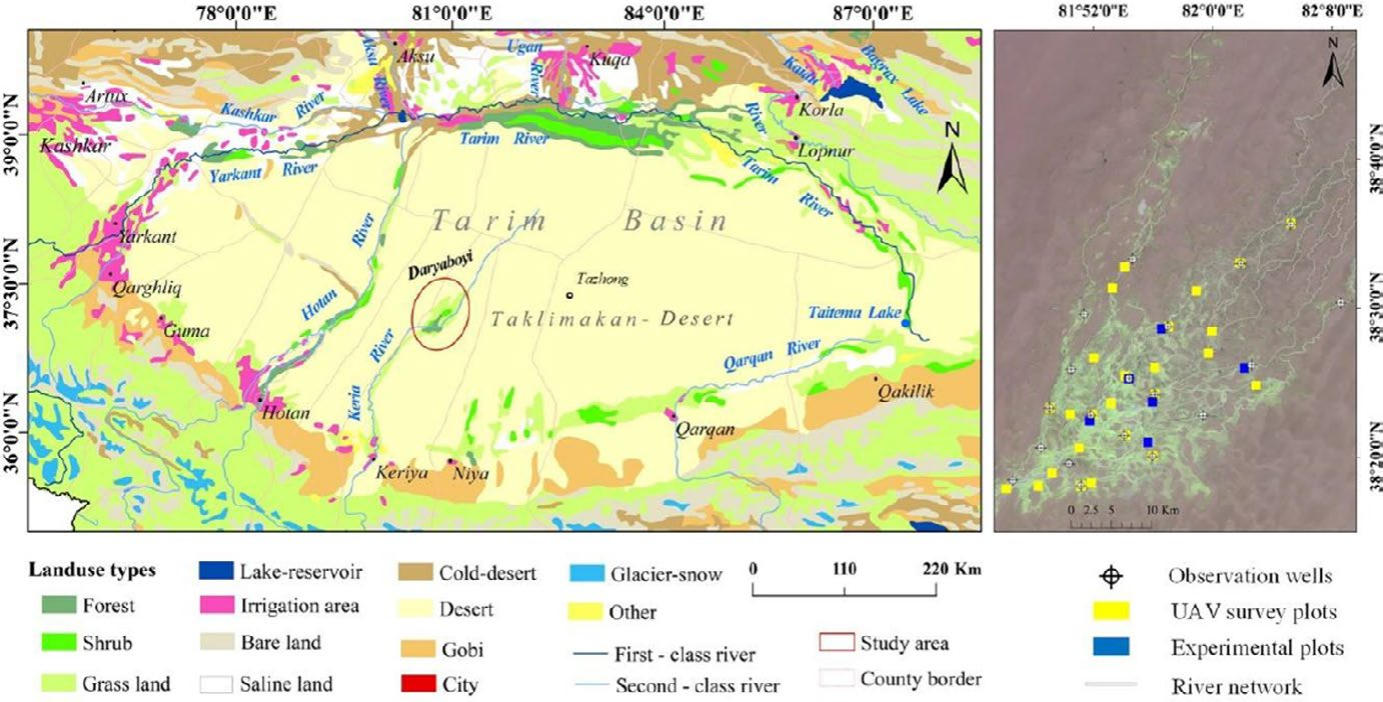
Sketch map of the study area and the locations of UAV vegetation surveying and experimental sampling sites

The Daryaboyi deltaic oasis in the lower reaches of the Keriya River is located in the center of the Taklimakan Desert at the upmost arid region of northwestern China (Figure 1). This oasis still retains its “primitive” state with a weak human interference, which is different from most existing modern oases in Central Asia (Shi et al., 2019; Zhang et al., 2011). The native vegetation types of this area are mainly dominated by *Populus euphratica* *(P. euphratica)* and *Tamarix ramosissima* *(T. ramosissima)*, which are considered to be groundwater‐dependent for maintenance of growth and ecological function (Hao et al., 2010; Li, Tong, et al., 2019; Li et al., 2010; Wu et al., 2016). These woody species play an irreplaceable role in protecting the development of oases, and maintaining the stability of riparian ecosystems, and the ecological functions of sand resistance and saline soil improvements (Chen et al., 2019; Wang et al., 2018; Zhang, Deng, et al., 2019). However, the expanding water demands for irrigation purposes in the up/midstream of Keriya River in the last decades have significantly decreased the river runoff into the downstream oasis. Consequently, the groundwater level has dropped significantly (Shi et al., 2019), which has caused a severe degradation of *Populus* and *Tamarix* communities in the oasis. In turn, the decline of the ecological function of riparian forests in Daryaboyi has resulted in the acceleration of desertification, petering out of river channels, and abandonment of settlements, especially in the northern part of the oasis. Over the past decades, tree populations have experienced severe drought, and almost 50% of the oasis area has been lost (Shi et al., 2019; Zhang, Zhou, & Nijat, 2019). Therefore, a scientific solution for the conservation and restoration of the two constructive desert riparian species in the Daryaboyi Oasis is urgently needed. However, very little is known about the water use strategies of dominant riparian species to different groundwater depth conditions. The main objectives addressed in this study are as follows: (a) to determine the desirable and stressing groundwater depths based on vegetation cover and plant growth status and (b) to examine the changes in water use strategies of plants in response to the groundwater depth gradient. From these perspectives, we hypothesized that the two plant species will not be subjected to water stress or salt stress under a desirable groundwater condition and will have a similar water use efficiency (δ^l3^C). Additionally, we also hypothesized that the water use efficiency of plants would increase with the decrease in water availability and result in higher foliar δ^13^C values.

## 2 MATERIALS AND METHODS

### 2.1 Trial area

The Keriya River Basin (35°14′–39°29′N; 81°09′–82°51′E) is located in the southern edge of the Tarim Basin and mid‐northern slope of the Kunlun (Karakorum) Mountains in Xinjiang Uyghur autonomous region, NW China (Figure 1). The climate is a typically arid continental climate with an average annual temperature of 11.6°C, precipitation of approximately 44.7 mm, and average evaporation of about 2,500 mm. The endorheic river basin consists of five prominent landforms of upstream mountains and piedmont Gobi, midstream alluvium and diluvium open area (Keriya Oasis), and downstream natural desert oasis—Daryaboyi, with the terrain declining from 5,460 to 1,180 m (Muyibul et al., 2018; Seydehmet et al., 2018).

The Daryaboyi Oasis (38°16′–38°37′N; 81°05′–81°46′E), the vanishing area of the Keriya River, is located in the hinterland of the Taklimakan Desert covering approximately 2,326.98 km^2^ (Figure 1). The oasis was formed by vessel‐shaped countless channel networks with three main river channels. The climate is hyperarid with annual precipitation of <10 mm (Zhang et al., 2011) and potential evaporation of 3,775 mm, which is almost 300 times greater than annual rainfall (Marhaba et al., 2020). In addition, the annual mean temperature is 12.1°C with the mean of minimum (Dec–Jan) and maximum temperature (July) of −17.07 and 36.26°C, respectively (referred to Tazhong station). The native vegetation is primarily drought‐resistant plants such as *Populus euphratica* Oliv, *Tamarix ramosissima*, and *Phragmites australis*, and other plants with a smaller distribution range. Sandy soil is the primary soil type in this area.

### 2.2 Experimental design and data acquisition

In mid‐August 2019, the vigorous growth period of dominant plants, twenty‐five unmanned aerial vehicle (UAV) survey images were collected to provide a broader assessment of the relationship between groundwater depth and vegetation cover. Besides, six focal experimental plots with different groundwater depth conditions were used to measure plant size, growth status, and leaf carbon content.

#### 2.2.1 Unmanned aerial vehicle vegetation coverage data

Unmanned aerial vehicle (UAV) vegetation survey plots (100 m × 100 m) were randomly selected within the research area according to the distribution patterns of dominant species (*P*. *euphratica* and *T*. *ramosissima*) and river system characteristics of the oasis (Figure 1). The DJI Phantom 4 (DJI‐Innovations Inc.) was used to obtain high‐resolution RGB images of vegetation survey sites such as the centimeter level with 80% overlap rate. The images were preprocessed using the photogrammetry software Pix4D mapper (Pix4D Inc.). Five vegetation indices were used to calculate the vegetation cover percentile value using the ENVI 5.3 (Esri Inc.), and the ExGR index was selected due to its high accuracy (Table S1).

#### 2.2.2 Selection of experimental plots

Experimental plots (100 m × 100 m), where *P*. *euphratica* and *T. ramosissima* coexist, were established according to the distribution patterns of dominant species and groundwater depth gradient (Figures 1 and S1). The growth status of each tree/shrub stands was defined according to the classification criteria reported by Aishan et al. (2015) based on the growth status (GS), including the degree of crown loss, leaves and branches, and canopy extension (Tables 1 and S2).

**TABLE 1:** Classification criteria of plant growth status

Degree	Growth status	Morphological characteristics
V1	Excellent	Crown almost or without signs of damage, that is, leaf loss ≤10%
V2	Good	Crown slightly damaged, that is, leaf loss 11%–25%
V3	Medium	Crown moderately damaged, that is, leaf loss 26%–50%
V4	Senesced	Crown heavily damaged, that is, leaf loss 51%–75%
V5	Dying	Tree almost strays, that is, leaf loss 76%–99%
V6	Dead	Fallen or no signs of vitality, that is, leaf loss 100%

Note

In this study, the GS of ≥ good state (V2) is referred to as a normal growth level.

In this study, the species' growth phase was grouped into three classes based on diameter at breast height (DBH) and crown diameter (CD), for example, for *P. euphratica* the DBH in the range of 0–10 cm (small‐sized plants), DBH in the range of 11–40 cm (medium‐sized plants), and DBH >40 cm (big‐sized plants); and for *T. ramosissima*, 0 < CD ≤ 2 m (small‐sized plants), 2 < CD ≤ 3 m (medium‐sized plants), and CD >3 m (big‐sized plants).

#### 2.2.3 Groundwater data

Groundwater data were obtained from 19 observation wells established in the sampling plots or the vicinity of the plots (Figure 1). The HOBO automatic monitoring device (Onset Computer Corporation) was used to measure and record the daily groundwater depths (6 times/day). Correspondingly, groundwater samples were taken in April and November (2019) from each observation well, and the measurement processes for the samples were carried out following the water quality analytical methods (SL78‐94‐1994). The chemical components in all the samples were tested in the Ecological and Environmental Analysis and Testing Center, Xinjiang Institute of Ecology and Geography, Chinese Academy of Sciences. The total dissolved solid (TDS) value is equal to the sum of major cations and anions. Ordinary kriging interpolation is a simple and accurate method commonly used to estimate the groundwater spatial distribution pattern (Ainiwaer et al., 2019; Ohmer et al., 2017; Xiao et al., 2016). Therefore, the groundwater depth data of UAV sampling plots were extracted using the Ordinary kriging geostatistical method by applying the ArcGIS software (Esri Inc., version 10.4). Groundwater depths of the area were classified into four levels: 0–2, 2–4, 4–6, and 6–8 m based on the depth gradient of observed and extracted water table data.

#### 2.2.4 Leaf sampling and carbon isotope analysis

In each experimental sampling plot, three to five tree/shrub stands with the same growing status were sampled, and leaves on the sunny side were collected. Only small‐ and big‐sized plants were discussed in this study due to their high sensitivity to water stress. All leaf samples were rinsed and air‐dried at room temperature (20°C), followed by oven‐drying at 60°C for 48 hr. Finally, oven‐dried leaves were ground to a powder and sifted through a 0.15‐mm sieve, and 0.5 g of the preprocessed powder from each leaf sample was used for test analysis. δ^13^C measurements were done using Delta V Advantage Isotope Ratio Mass Spectrometer (Thermo Fisher) in Xinjiang Institute of Ecology and Geography, Chinese Academy of Sciences. The carbon isotope abundance (δ^13^C, ‰) was expressed as the isotopic ratio of a sample relative to the Pee Dee Belemnite (PDB) standard calculated using Equation (1) (Qin et al., 2020):

(1) $$\delta^{13}C\left(‱\right)=\left[\frac{R_{\text{sample}}‐R_{\text{standard}}}{R_{\text{standard}}}\right]\times 1000$$

where *R*_sample_ is the isotopic ratio of ^13^C_sample_/^12^C_standard_, and *R*_standard_ is that of PDB standard.

### 2.3 Data processing

Statistical analyses and data visualization were performed using the Microsoft Excel 2016 and OriginPro 2020 (Origin Lab Inc.). One‐way analysis of variance (ANOVA) and Duncan's test were carried out using the SPSS 21.0 (IBM) to assess the significant differences between δ^13^C values of same plant species with different groundwater depths. Simultaneously, the Dunn–Sidak test was performed using the Paired Comparison Plot APP, OriginPro 2020 (Origin Lab Inc.), to determine a significant difference between δ^13^C values of two species at the same groundwater depth.

## 3 RESULTS

### 3.1 Difference in vegetation characteristics related to groundwater depth gradient

**FIGURE 2:**
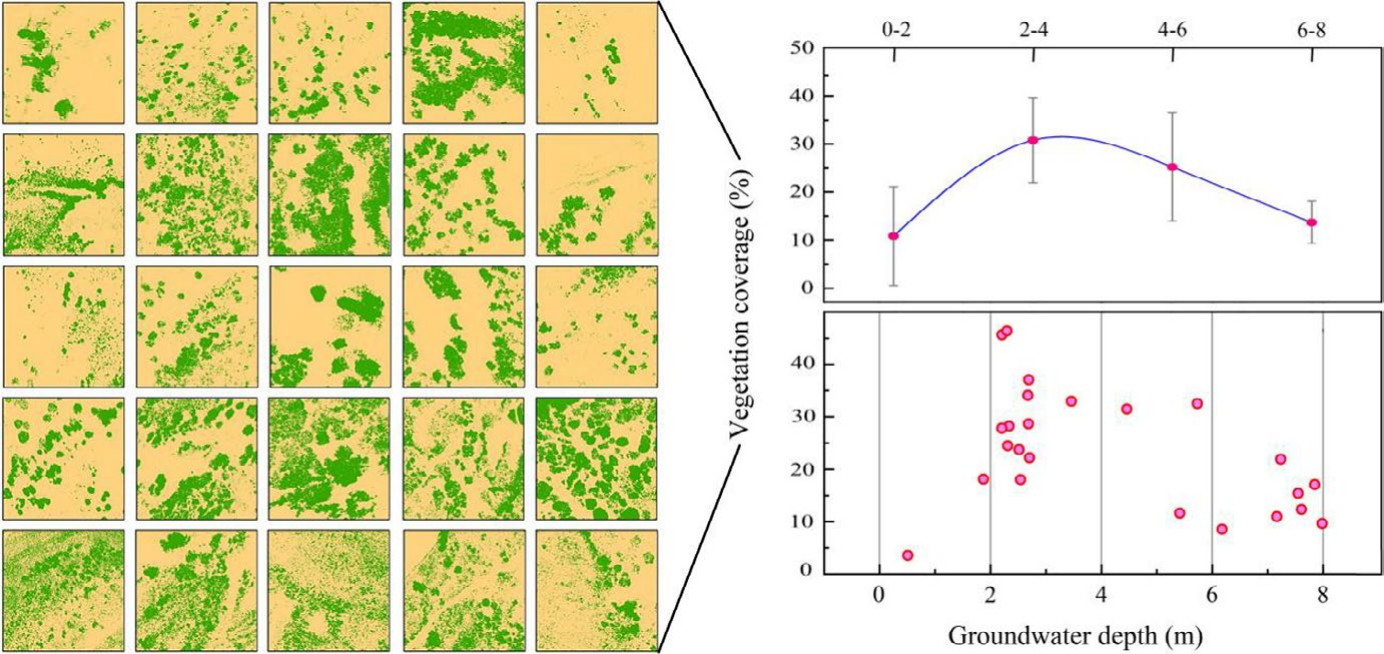
Vegetation cover ratios under different groundwater depths in the Daryaboyi Oasis. In UAV fractional vegetation cover images, the green color represents vegetation, and the dark yellow represents bare land

Unmanned aerial vehicle (UAV) is a new platform that has been widely used for forestry applications because of its many advantages, including availability, low budget, reliability, autonomy, and timely high‐resolution data (Akturk & Altunel, 2019). The obtained results indicated that the fractional vegetation coverage (FVC) values of the plots ranged from 4% to 18% when the GWD was <2 m, with an average value of 10.84% (Figure 2). However, the highest average FVC value (30.79%) was observed at GWDs of 2*–*4 m, and the values varied from 18% to 45%. The average value declined to 25.21% at GWDs of 4–6 m, with the changing range of 11%–33%. The FVC values of the plots ranged between 9% and 22% when the GWD was 6*–*8 m, and the average coverage was lower than 20%. Overall, the average coverage ratios of dominant species, mainly *P*. *euphratica* and *T. ramosissima*, in different plots exhibited a decreasing trend when GWD interval increased from 2*–*4 m to 6–8 m or decreased to 0–2 m.

The “tree growth status” measurement can be used as a crucial indicator for assessing a forest ecosystem's health conditions, integrity, and resilience (Halik et al., 2019). The differences in tree growth status in the six mixed plant plots (mainly consisting of *P*. *euphratica* and *T. ramosissima*.) along the groundwater availability gradients were grouped and are summarized in Table S1. For *P*. *euphratica*, small‐sized plants grew in a normal state at GWDs of 2.1–4.3 m, and significant numbers of dead stands were observed when the GWD was 5.7 m. In addition, medium‐sized plants could not grow normally at 6.9 m (belongs to the medium growth status) and were subjected to senesce when the GWD dropped to 7.8 m. Besides, big‐sized plants grew normally at GWDs ranging from 2.1 to 6.7 m, with dead twigs appearing at 7.8 m. For *T*. *ramosissima*, results obtained from field investigation indicated that small‐sized plants belong to excellent growth level were observed at GWDs of 2.1–4.3 m, whereas large proportions of small‐sized plants were severely damaged or dead at GWD ≥5.7 m. In addition, the crown of middle‐sized *T. ramosissima* plants were moderately damaged at 7.8 m, while big‐sized plants were in an excellent or normal state when the GWD was between 2.1 and 7.8 m.

### 3.2 Changes in δ^13^C values of *P. euphratica* along with groundwater depth gradient

**FIGURE 3:**
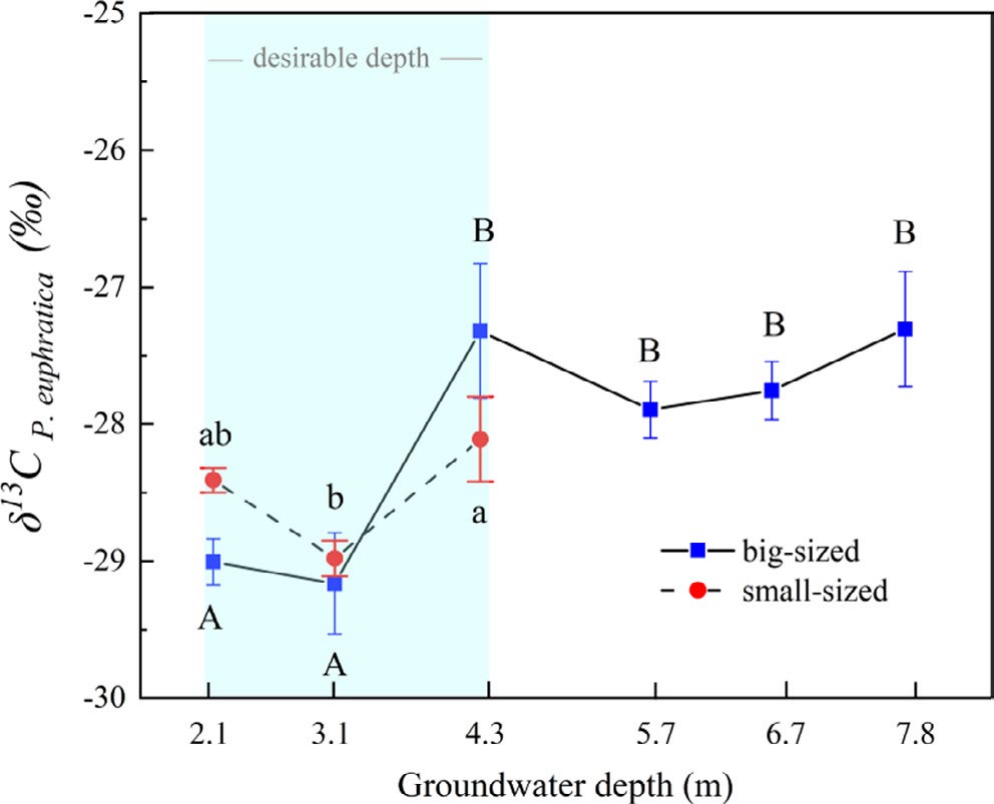
Carbon isotope content of *P. euphratica* leaves of small‐ and big‐sized plants. Different letters indicate significant differences (*p* < .05) between δ^13^C values of *P*. *euphratica* at different groundwater depths and vice versa (capital letters for big‐sized plants and lowercase letters for small‐sized plants)

Leaf carbon isotope content is the key indicator that reflects the plant water use efficiency in arid regions. Evident changes were observed in leaf δ^13^C values of *P. euphratica* at different groundwater burial depths (Figure 3). Although the δ^13^C values of small‐sized plants initially decreased to its lowest value of −28.98‰ at 3.1 m and sharply increased to a maximum value of −28.11‰ at 4.3 m, there were no significant differences between δ^13^C values at 2.1‐ and 3.1‐m and at 2.1‐ and 4.3‐m GWD. However, the δ^13^C values differ measurably when the GWD increased from 3.1 m to 4.3 m (*p* < .05). For big‐sized *P. euphratica* plants, the leaf δ^13^C values ranged from −29.01 to −29.16‰ at GWDs ranging from 2.1 to 3.1 m, and there were no significant changes in water use efficiency at this stage (*p* < .05). Interestingly, the δ^13^C value increased sharply and peaked at −27.07‰ when the GWD was 4.3 m, which was significantly higher than the δ^13^C value of GWDs at 2.1–3.1 m (*p* < .05). The differences between the leaf δ^13^C value *in P. euphratica* at GWDs ranging from 4.3 to 8.7 m were not significant despite the value initially decreasing followed by a gradual increase (*p* < .05).

### 3.3 Changes in δ^13^C values of *T. ramosissima* along with groundwater depth gradient

**FIGURE 4:**
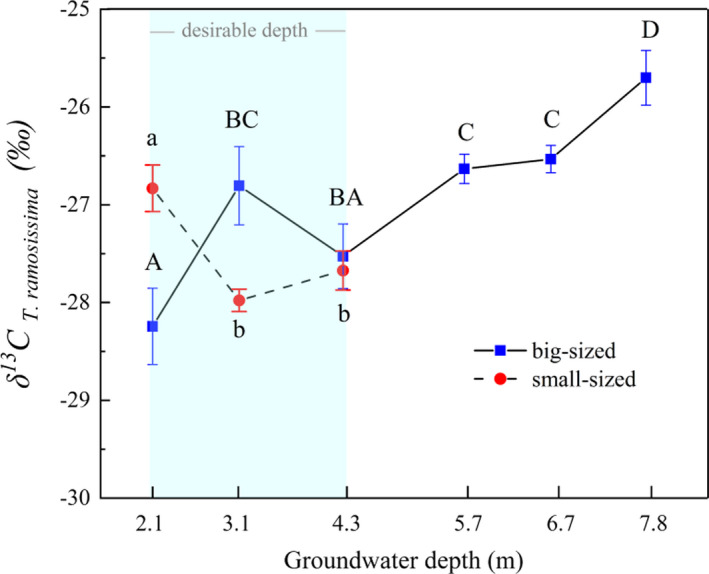
Carbon isotope content of *T. ramosissima* leaves for small‐ and big‐sized plants. Different letters indicate significant differences (*p* < .05) between δ^13^C values of *T. ramosissima*. at different groundwater depths and vice versa (capital letters for big‐sized plants and lowercase letters for small‐sized plants)

Figure 4 shows the response of the δ^13^C amount in leaves of small‐ and big‐sized *T. ramosissima* plants to the groundwater depth gradient. For small‐sized plants, the leaf δ^13^C value at 2.1‐m GWD was higher than that at GWDs ranging from 3.1 to 4.3 m (*p* < .05), and almost the same degree δ^13^C values of the big‐sized plants at GWDs between 5.7 and 6.7 m. This indicates that the water use efficiency of *T. ramosissima* at 2.1 m may have been affected by other influencing factors. There was no significant change in water use efficiency of *T. ramosissima*. at 3.1‐ to 4.3‐m GWD (*p* < .05). For big‐sized plants, the water use efficiency of *T. ramosissima* sharply increased when the GWD increased from 2.1 m to 3.1 m and was almost 2.0‰ higher than that at 2.1‐m GWD. Although a minor difference was observed for δ^13^C values at GWDs ranging from 5.7 to 6.7 m, the overall water use efficiency of *T. ramosissima* increased significantly when the GWD varied from 4.3 to 8.7 m (*p* <.05).

### 3.4 Comparison of δ^13^C values in *P. euphratica* and *T. ramosissima* at different groundwater depth

**FIGURE 5:**
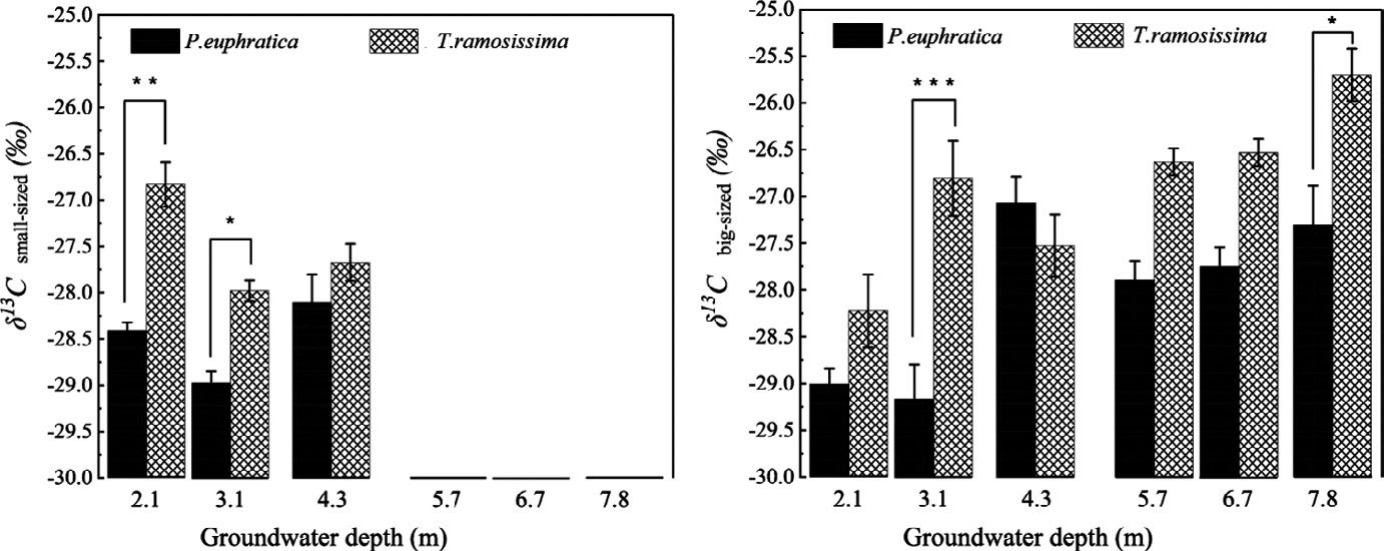
Difference in δ^13^C value in leaves of small‐ and big‐sized *P. euphratica* and *T*. *ramosissima* plants grown at different groundwater depths. The asterisk (*) indicates a significant difference between δ^13^C values of two plant species at the same groundwater depth (*p ≤ .05, **p ≤ .01, and ***p ≤ .001)

For the small‐sized plants grown at 2.1‐m GWD, *T. ramosissima* had a higher δ^13^C value than at the other two sites, while the δ^13^C value in *P. euphratica* fell in between GWDs of 3.1 m and 4.3 m (Figure 5). Besides, the δ^13^C values in *T. ramosissima* (−26.83‰) were significantly higher when compared to the corresponding δ^13^C values in *P. euphratica* (−28.41‰) at GWDs of 2.1–3.1 m (*p* ≤ .01 and *p* ≤ .05, respectively). The insignificant difference in leaf δ^13^C values between *P. euphratica* and *T. ramosissima* at 4.3‐m GWD suggests that these two species had the same water use efficiency or similar water use strategies.

For big‐sized plants, leaf δ^13^C values of both plant types responded differently to the changes in groundwater depth when the groundwater depth was <4.3 m (Figure 5). The δ^13^C value in *T. ramosissima* was remarkably higher than that in *P. euphratica* when the groundwater depth was 3.1 m (*p* < .001), whereas an insignificant difference was observed at 2.1‐m GWD. Surprisingly, the δ^13^C value in *P. euphratica* at a depth of 4.3 m slightly exceeded the corresponding *T. ramosissima* δ^13^C value and was almost the same degree with *P. euphratica* δ^13^C value at 7.8 m. However, when the GWD was between 5.7 and 7.8 m, the δ^13^C values of both plants increased with the increase in groundwater depth gradient, and the δ^13^C value of *T. ramosissima* was significantly higher than the corresponding *P. euphratica* value (about 1.31‰) (*p* ≤ .05). The above results indicate that the overall water use efficiency of *T. ramosissima* is higher than that of *P. euphratica* in both small‐ and big‐sized plants.

## 4 DISCUSSION

### 4.1 Responses of vegetation characteristics to the groundwater condition

Groundwater is the primary and steady water source for riparian forests in the Daryaboyi Oasis due to the sparse precipitation and limited surface water flow. Studies have reported that maintaining the desirable groundwater depth is vital for sustaining the function and healthy growth of xerophyte communities (Lang et al., 2016; Zhu et al., 2013). Desirable groundwater depth is defined as the limits of groundwater depths that satisfy the physiobiological demand for growth of natural vegetation without water stress caused by water deficiency or water salinity derived from higher groundwater level (Huang, Zhang, & Chen, 2019; Huang, Zhang, Zhang, et al., 2019; Wang et al., 2019). In this study, the probable desirable groundwater depth was estimated based on obtained UAV vegetation cover images combined with plant growth state (Table S1). The results indicated that the average FVC value was highest (30.79%) at GWDs ranging from 2 to 4 m, and it declined to 25.21% at GWDs of 4–6 m. The lowest FVC values of less than 20% were obtained when the GWD was >6 m or <2 m. This result was consistent with a study conducted downstream of the Tarim River (Hao et al., 2010). Tree vitality is a crucial indicator for assessing a forest's health conditions, integrity, and resilience (Halik et al., 2019). For both *P. euphratica* and *T. ramosissima*, small‐sized plants grew normally only when the GWD was between 2.1 and 4.3 m, and the vitality of medium‐sized plants declined from its normal state to a severely damaged level when the water depth was deeper than 5.7 m and 6.7 m, respectively. However, a GWD of ≥7.8 m was not suitable for the normal growth of big‐sized *P*. *euphratica* plants, but *T. ramosissima* still grew in a normal state even when the water depth was 7.8 m. The above results indicate that the desirable groundwater depth should be kept at about 2.1–4.3 m when considering the normal growth stats of these two plant species at three size classes, which will support their survivability and regeneration. These findings are consistent with the results of previous studies conducted on the Lower Tarim River region. Ma et al. (2011), Chen et al. (2013), and Halik et al. (2019) reported that the desirable ecological water level for *P*. *euphratica* and *T. ramosissima* in Lower Tarim River region should be below 4 m, and a water depth ≥9 m may cause partial or total senescence of *P. euphratica* trees. The studies also reported that the suitable groundwater depth for survivability of *P*. *euphratica* in Lower Tarim River region is 2–4 m, and the threshold depth is 7–8 m. The maximum biomass of *Tamarix* was observed when the GWD was <4.5 m, and it significantly decreased when the GWD was >7 m (Chen et al., 2015; Han et al., 2017; Hao et al., 2010; Li et al., 2010). Therefore, the actual groundwater depth should be kept at a range about 2.1–4.3 m, and the minimum (threshold) groundwater depth should be less than 7 m. This will help in the protection of riparian woody plants at normal growth state and will guarantee the coexistence of both plant types.

### 4.2 Variations of plant water use efficiency under the desirable groundwater condition: effects of water alternation

**FIGURE 6:**
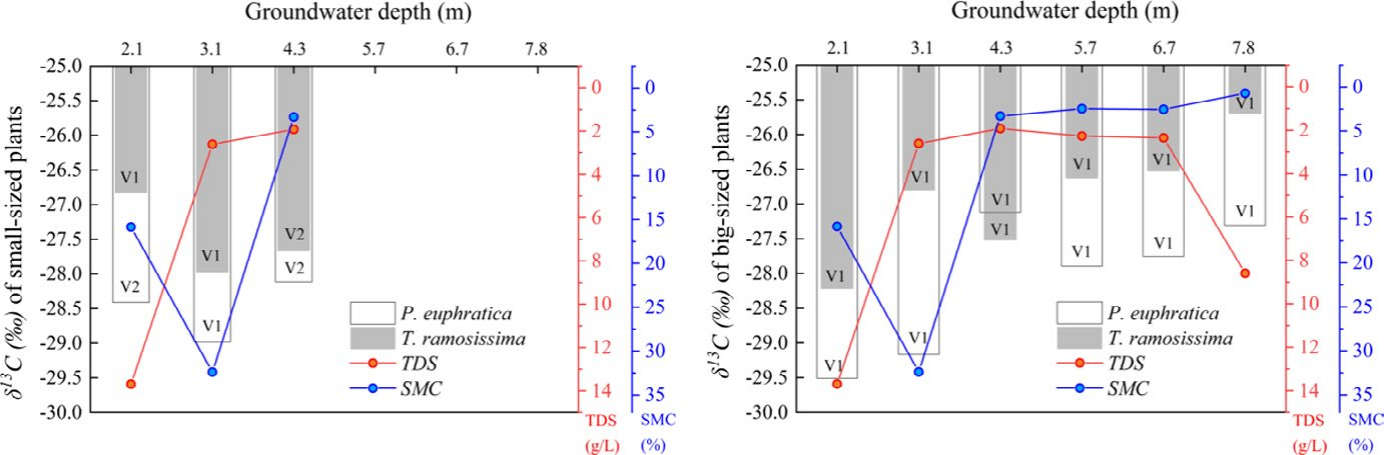
Foliar δ^13^C, plant growth degree (V, Table 1), TDS (total dissolved solid), and SWC (subsurface soil water content from 50‐ to 100‐cm soil layer) values of experimental plots under different groundwater table depths

Water stress can limit plant growth more significantly than any other factor (Si et al., 2015). Foliar δ^13^C can be used as a proxy for assessing the plant's physiological response to water stress within or interspecies. It is also an essential indicator for demonstrating plant water use efficiency due to the strong positive correlation between them (Cao et al., 2020). It has been reported that desert plant species resist water stress by improving water use efficiency (Marhaba et al., 2020). A parallel study in the lower reaches of Tarim River reported that plants of the same species or functional group will not be subjected to water stress and may have similar δ^13^C values when growing under a desirable groundwater condition (Ren et al., 2014). In this study, the foliar δ^13^C of *P*. *euphratica* and *T. ramosissima* responded differently to the changes in water depth when the GWD was between 2.1 and 4.3 m. The δ^13^C value of big‐sized *T*. *ramosissima* (−27.89‰) was slightly higher than that of *P. euphratica* (−28.66‰) when the depth was 2.1 m, and the values were not significantly different (*p* > .05) (Figure 5). However, small‐sized *T. ramosissima* plants experienced a higher δ^13^C value at a significant level (*p* < .05) than those at 3.1‐m and 4.3‐m GWD (Figure 6), and the value was also significantly higher than the corresponding *P. euphratica* value (*p* ≤ .01). Ye et al. (2009), Huang, Zhang, and Chen (2019) and Huang, Zhang, Zhang, et al. (2019) reported that riparian plants could suffer from anoxia or salt stress when the groundwater level is too high, thereby harming plant mortality. In addition, Zhang, Zhou, and Nijat (2019) reported that salinity influences the distribution of *P*. *euphratica* by restricting the growth of stands and germination of seeds. Figure 6 shows that the TDS exhibited a trend of first decreasing and then increasing with an increase in the groundwater depth, and it reached its maximum when the groundwater depth was 2.1 m. In our field investigation, we found that *P. euphratica* trees begin to die from top to stem base, and small‐sized plants have died out, whereas *T*. *ramosissima* still maintain its normal growth when the GWD is about 1.5 m (Figure S2). A previous study also reported that TDS up to 17.55 g/L could severely restrict WUE of *P*. *euphratica* saplings, resulting in a severe water deficit in the leaves and a sharp reduction in water transport via the xylem (Zhang, Deng, et al., 2019; Zhou et al., 2020). The above results combined with the growth status (Figure 6) indicate that small‐sized plants in both plant species might suffer from salt stress induced by shallow GWD. In response, the plants resist this stress through different adaptive strategies such as reducing growth status in *P*. *euphratica* (Figure 6) and increasing water use efficiency by *T. ramosissima*. The obtained results in this study also suggest that big‐sized plants are more salt‐tolerant than the small‐sized plants in the riparian ecosystem, and *T. ramosissima* has stronger salt tolerability than *P*. *euphratica*. These results are consistent with Li et al. (2013) and Zhang, Deng, et al. (2019).

Interspecific competition is more likely to occur under high water availability, thereby leading to the plants achieving rapid growth (Wu et al., 2016). Under a desirable groundwater depth, the water alternation induced by different recharging methods such as flooding and rising groundwater depth may cause competition between *P. euphratica* and *T. ramosissima* to access water resources for occupying the riparian zones, which results in an increase or decrease in leaf δ^13^C. Studies have reported that flooding is the precondition for *P*. *euphratica* growth, regeneration, and restoration, mainly due to the pivotal role of recharging soil water and reducing soil salinity (Wu et al., 2018; Zhang, Deng, et al., 2019). In this study, the sampling site where GWD was 3.1 m had been flooded a week before field investigation, and thus, a high soil water content (Figure 6) with a comparatively lower TDS in groundwater (Figure 6) was observed. A significant difference in δ^13^C values between *P. euphratica* and *T. ramosissima* at 3.1 m in both size groups (*p* ≤ .05 for small‐sized plants and *p* ≤ .001 for big‐sized plants) can be attributed to the fact that *P. euphratica* growth is favored after an area has suffered flooding disturbances, and this may lead to the dominance of *P. euphratica* in the riparian plant communities. Although the δ^13^C values of the two species were not significantly different (*p* ≤ .05), the leaf δ^13^C content of *P. euphratica*, surprisingly, exceeded *T. ramosissima* when the GWD was 4.3 m. Wu et al. (2018) and Li, Tong, et al. (2019) have reported that *T. ramosissima* benefits from its rapid root system responses to groundwater alterations, which allows it to access groundwater earlier than *P. euphratica* once the groundwater depth is raised. Steinberg et al. (2020) found that *Tamarix* cover will increase with intra‐ and interannual variation in groundwater depth. During the field investigation, we found a river channel with a small water flow located 20 m away from the sampling site, which is the probable recharging source for the groundwater. The high δ^13^C value of *P. euphratica* in this study could be due to the competition by the plants for groundwater alternation, which favors *T. ramosissima* over *P. euphratica*. However, no significant sensitive response was found for the small‐sized plant species at 4.3‐m GWD. Generally, the anomalies in δ^13^C values of woody plant species in a desirable groundwater depth range are more likely associated with salt stress and interspecific competition caused by water alternation. The long‐term effect of these factors requires further investigation.

### 4.3 Water use strategies of plants to the decreased water availability revealed by differences in δ^13^C and plant growth status

Riparian trees may experience water‐deficit stress and reduce productivity as the water table accessibility is reduced (Pettit and Froend, 2018). Correspondingly, plants respond adaptively to external stress conditions by applying internal adjustment mechanisms to resist drought stress induced by extreme or unexpected water shortage (Chen et al., 2013). In drought conditions, *P*. *euphratica* photosynthesizes using both sides of leaves to improve water use efficiency and effectively controls transpiration loss by adjusting stoma conductance (Ren et al., 2014). On the other hand, *T. ramosissima* maintains a relatively high transpiration rate to avoid severe drought stress even in instances where the groundwater depth is more in‐depth than for *P*. *euphratica* (Marhaba et al., 2020). In general, the capacity of *P. euphratica* to maintain hydraulic balance was significantly weaker than that of *T. ramosissima*, and severe drought may lead to branch dieback in *P*. *euphratica* faster than in *T. ramosissima* (Li, Si, Zhang, Gao, Luo, et al., 2019). To adapt to severe drought, *T. ramosissima* utilizes an optimal water absorption strategy and shifts the water source to obtain more stable moisture, which was beneficial to *T. ramosissima* to enhance hydraulic efficiency in the ecosystem (Su et al., 2020). However, *P. euphratica* improves the hydraulic conductance of branches with minor hydraulic limitation and diminishes branches with higher hydraulic limitation to avoid total loss of internal hydraulic regulation (Li, Si, Zhang, Gao, Wang, et al., 2019). This study found that the change in groundwater depth resulted in a difference between the δ^13^C values of *P. euphratica* and *T. ramosissima* when the GWD was between 5.7 and 7.8 m. Besides, the two plant species exhibited different growth states and water use strategies in the same groundwater condition. For example, *P*. *euphratica* maintains its normal growth condition at a GWD between 5.7 and 6.7 m mainly by decreasing aboveground biomass, thereby declining growth conditions (from excellent to good). At the same time, *T. ramosissima* maintains its normal state by increasing water use efficiency. However, when the GWD was 7.8 m, *P*. *euphratica* eliminated more branches to keep intrinsic water use efficiency, and *T. ramosissima* maintained its life condition by using adaptive strategies to increase water use efficiency first and decrease health status after then (Figure 6). Results also showed that *T. ramosissima* has strong adaptability to drought conditions than *P*. *euphratica* has.

## 5 CONCLUSIONS

In this study, water use strategies of dominant riparian woody plant species were identified along a gradient of groundwater depth in the lower reaches of the Keriya River–Daryaboyi Oasis. The following results were obtained in the study:

1. Captured UAV‐based vegetation cover images and field‐observed plant growth status data indicated that the groundwater depth should be kept in a desirable range of about 2.1–4.3 m to support the survivability, regeneration, and coexistence of *P*. *euphratica* and *T. ramosissima* communities. In addition, the threshold groundwater depth should be smaller than 7 m to guarantee normal health status of overmature stands for both plant species.
2. Contrary to our hypotheses, the leaf carbon isotopic contents of the two plant species showed flexible patterns in the three sampling sites where groundwater depths were appropriate. When the groundwater was 2.1–4.3 m, salt stress and interspecific competition caused by water alternation, including flooding and rises in groundwater depth, led to the significant differences in leaf δ^13^C values of *P*. *euphratica* and *T. ramosissima*. The study also found that big‐sized plants are more salt‐tolerant than seedlings or small ones, and *T. ramosissima* has strong salt tolerability than *P*. *euphratica*.
3. Inconsistent with our hypotheses, *P*. *euphratica* generally utilizes a pure strategy with the reduction in water availability to withstand drought stress, while *T. ramosissima* adopts multiple solutions. The study also found that *T. ramosissima* has strong adaptability to drought conditions than *P. euphratica*.

The aim of this study was restricted by the availability of enough sampling tree/shrub stands, generally three to five, of different sizes in each experimental plot. Consequently, we were not able to discuss the response of medium‐sized plants to the changes in groundwater depths, although they were recognized as the main composition of the plant communities. Therefore, further study on the response mechanisms of plants of all three sizes to the long‐term dynamical changes of groundwater depth will be critical in the future.

## CONFLICT OF INTERESTS

The authors declare that they have no conflict of interest.

## AUTHOR CONTRIBUTIONS

**Bilal Imin:** Data curation (equal); Software (equal); Visualization (equal); Writing‐original draft (lead). **Dai Yue:** Formal analysis (equal); Methodology (equal). **Qingdong Shi:** Funding acquisition (supporting); Methodology (equal). **Yuchuan Guo:** Investigation (equal); Supervision (equal). **Hao Li:** Investigation (equal); Software (equal). **Marhaba Nijat:** Data curation (equal).

## Supporting information

Supplementary Material

## Data Availability

All the laboratory experimental data in this study are available from the Dryad Digital Repository: https://doi.org/10.5061/dryad.t4b8gtj0m.
