# Supplementary material for: Responses of two dominant desert plant species to the changes in groundwater depth in hinterland natural oasis, Tarim Basin
Source: Ecol Evol. 2021 Jun 16;11(14):9460–71. doi: 10.1002/ece3.7766 (PMC8293730; doi:10.1002/ece3.7766)
Supplement: Supplementary file 1 — Supplementary Material [file ECE3-11-9460-s001.docx]

**Supporting information**

**TABLE S1** UAV image (RGB)-based vegetation coverage indices applied in this study.

| Name | Equation | Reference |
| --- | --- | --- |
| GRVI (Green red vegetation index) | ${G-R}/{G+R}$ | (Yeom *et al.*, 2019) |
| ExGI (Excess green index) | ${2G}_{n}-R_{n}-B_{n}$ | (Yeom *et al.*, 2019) |
| ExGRI (Excess green minus excess red) index) | $ExGI-1.4R_{n}-G_{n}$ | (Yeom *et al.*, 2019) |
| VegI (Vegetive index) | $G/\left( R^{0.667}+B^{0.333} \right)$ | ( Li *et al.*, 2019c) |
| ExRI (Excess red index) | $1.4R-G$ | (Li *et al.*, 2019c) |

**Note:** R, G, B represents red, green, and blue bands. R_n_ = R/(R+G+B), G_n_ = G/(R+G+B), B_n_ = B/(R+G+B).

**TABLE S2** The tree/shrub stand parameters and growth status of species found at the Daryaboyi Oasis.

| **Plant types** | **GWD (m)** | **H(m)** | **DBH (cm)** | | **CD (m)** | **GS** | | |
| --- | --- | --- | --- | --- | --- | --- | --- | --- |
|  |  |  |  |  |  | **young** | **mature** | **over-mature** |
| ***Populus euphratica*** | 2.1 | 5.4 | | 16.9 | 3.83×3.04 | V2 | V1 | V1 |
|  | 3.1 | 5.40 | | 39.7 | 3.19×2.75 | V1 | V1 | V1 |
|  | 4.3 | 5.39 | | 32.4 | 3.82×3.51 | V2 | V2 | V1 |
|  | 5.7 | 6.58 | | 43.7 | 4.72×4.38 | V5 | V2 | V2 |
|  | 6.7 | 6.63 | | 44.7 | 4.99×4.17 | — | V3 | V2 |
|  | 7.8 | 5.74 | | 46.6 | 4.71×4.20 | — | V4 | V3 |
| ***Tamarix spp.*** | 2.1 | 1.33 | | — | 1.28×1.11 | V1 | V1 | V1 |
|  | 3.1 | 1.67 | | — | 1.02×0.96 | V1 | V1 | V1 |
|  | 4.3 | 1.68 | | — | 1.55×1.47 | V2 | V1 | V1 |
|  | 5.7 | 1.25 | | — | 1.35×1.08 | V4 | V2 | V1 |
|  | 6.7 | 1.97 | | — | 1.51×1.40 | — | V2 | V1 |
|  | 7.8 | 2.02 | | — | 1.56×1.38 | — | V3 | V2 |

**Note:** V1: excellent, V2: good, V3: medium, V4: senesced, V5: dying, and V6: dead. The vitality of ≥ a good state (V2) is referred to as a normal growth level.

**FIGURE S1** Changes in groundwater depth during plat vigorous growth period.


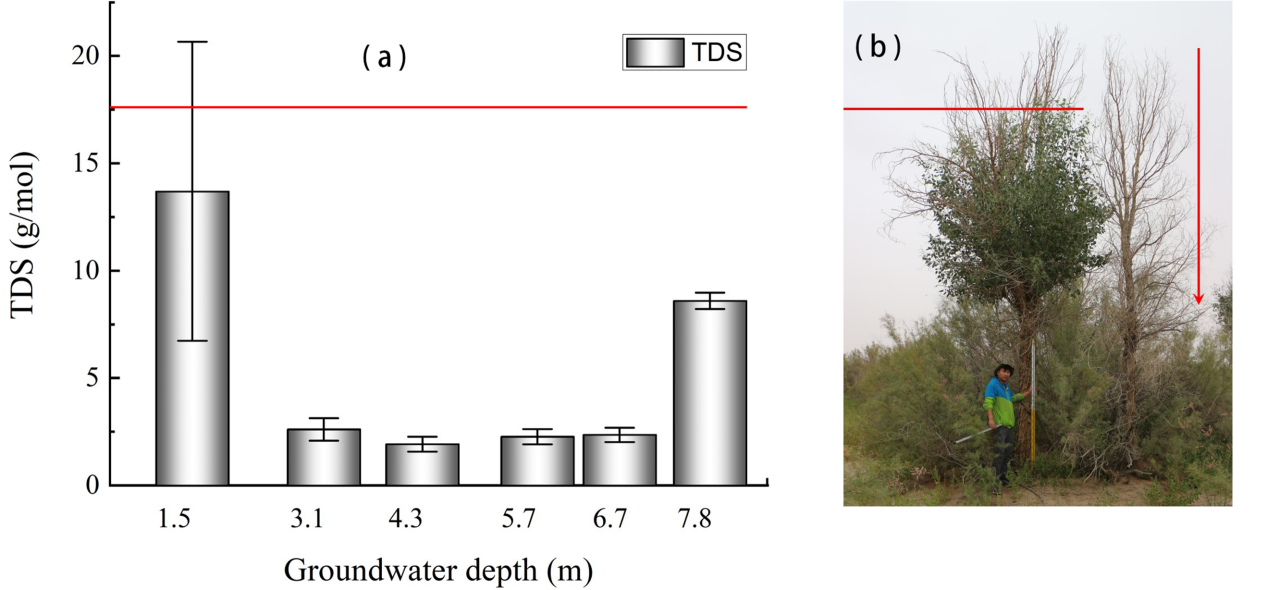


**FIGURE S2** The TDS values of survey plots under different groundwater table depths (a). The phenomenon of tree death from top to stem base (b). The filed survey plot where GWD was 1.5 m was almost 3Km away from the sampling site with a GWD of 2.1m.
